# Efficacy of traditional Chinese medicine combined with Silibinin on nonalcoholic fatty liver disease: A meta-analysis and systematic review

**DOI:** 10.1097/MD.0000000000037052

**Published:** 2023-02-02

**Authors:** Xiang Zhang, Zhenghao Jiang, Xiaoliang Jin, Qiujun Zhou

**Affiliations:** a The First Affiliated Hospital of Zhejiang Chinese Medical University (Zhejiang Provincial Hospital of Chinese Medicine), Hangzhou, China; b Department of First Clinical Medical College, Zhejiang Chinese Medical University, Hangzhou, China.

**Keywords:** efficacy, meta-analysis and systematic review, nonalcoholic fatty liver disease, Silibinin, traditional Chinese medicine

## Abstract

**Background:**

The efficacy and safety of traditional Chinese medicine (TCM) combined with Silibinin in the treatment of nonalcoholic fatty liver disease (NAFLD) are still inconclusive. This meta-analysis intends to evaluation to explore the clinical efficacy and quality assessment of traditional Chinese medicine in combination with Silymarin in the treatment of NAFLD, aiming to aims to provide evidence-based data analysis for researchers and clinical practitioners involved in TCM research for NAFLD, with the hope of facilitating wider adoption and application

**Methods:**

In this meta-analysis, we searched PubMed, Embase, Cochrane Library, CNKI, Wanfang, CQVIP and CBM databases from the establishment of the databases to Oct 2023. The study proposed to include studies that reported combination of TCM with Silibinin and Silibinin alone in the treatment of NAFLD, excluding studies for which full text was not available or for which data extraction was not possible; studies using animal studies; reviews and systematic reviews. All data were processed by STATA15.1 statistical software.

**Results:**

16 randomized controlled trials (RCTs) were included in this meta-analysis. The sample size ranged from 48 to 120, with a total of 1335 patients, including 669 in the Combined treatment group and 384 in the Silibinin group. The findings indicated that the total effective rate of combined treatment group was significantly higher than that of Silibinin alone. Levels of alanine aminotransferase (ALT), aspartate aminotransferase (AST), total cholesterol (TC), triglycerides (TG), and gamma glutamyl transpeptidase (GGT) of combined treatment group were all significantly lower than that of western medicine alone. Additionally, after treating NAFLD with a combination of TCM and Silibinin, the TCM syndrome score were significantly lower than those observed with Silibinin alone.

**Conclusion:**

Traditional Chinese medicine in conjunction with Silibinin capsules has shown significant efficacy in the treatment of NAFLD, improving clinical symptoms, blood lipid levels, and liver function. Furthermore, it is essential to engage in multi-omics research, investigate iron death, and explore the gut microbiota as potential observational indicators for the diagnosis and inclusion criteria. Conducting more high-quality clinical experiments is necessary to further validate these findings.

## 1. Introduction

nonalcoholic fatty liver disease (NAFLD) is a prevalent clinical manifestation of metabolic syndrome, primarily distinguished by the accumulation of excessive fat in the liver, defined as more than 5% fat content.^[1,2]^ Importantly, NAFLD occurs independently of autoimmune factors, drug-induced effects, viral hepatitis, or a history of excessive alcohol consumption.^[3]^ NAFLD can progress from nonalcoholic simple fatty liver to nonalcoholic steatohepatitis, liver fibrosis, and even advance to liver cirrhosis and hepatocellular carcinoma.^[4]^ Cirrhosis is a late-stage organ failure of the liver that often requires a liver transplant, and liver cancer typically develops decades after cirrhosis or chronic hepatitis B infection. Both of these conditions are common causes of liver disease-related deaths in patients.^[5,6]^ According to statistics, the global prevalence of NAFLD in the general population has reached 25%, with rates among Asian populations ranging from 15% to 40%.^[7]^

Research has shown that insulin resistance and genetic susceptibility play a role in the development of this disease.^[8]^ Insulin resistance can promote the accumulation of lipids in the liver, causing the initial impact on the liver. The accumulated lipids can further trigger events such as inflammation and oxidative stress, leading to cellular toxicity and damaging liver function, constituting the ‘two-hit’ theory recognized by many scholars in the pathogenesis of nonalcoholic fatty liver disease.^[9]^ At present, Silibinin capsules are commonly used in clinical treatment for nonalcoholic fatty liver disease. They can eliminate reactive oxygen species within liver cells, facilitate detoxification, and subsequently promote liver function recovery. However, using them as a standalone treatment may not achieve the desired therapeutic effect. The pathogenesis of NAFLD is complex, and the disease progression is extensive. While there has been some progress in drug therapy, there is still no specific medication approved for the treatment of NAFLD. Traditional Chinese medicine (TCM) has gained increasing attention in the treatment of NAFLD, as it offers a unique advantage with its multi-component and multi-target effects in liver disease therapy. In recent years, multiple studies have indicated that a combination of traditional Chinese and Western medicine in treating NAFLD is more effective than Western medicine alone in improving abnormal liver function, reducing blood lipids, and enhancing insulin resistance, among other aspects.^[10,11]^ Based on this, our study includes randomized controlled trials of traditional Chinese medicine in combination with Silymarin capsules for the treatment of NAFLD in accordance with the standards. We intend to use meta-analysis and systematic evaluation to explore the clinical efficacy and quality assessment of traditional Chinese medicine in combination with Silymarin in the treatment of NAFLD. This research aims to provide evidence-based data analysis for researchers and clinical practitioners involved in TCM research for NAFLD, with the hope of facilitating wider adoption and application.

## 2. Methods

### 2.1. Literature inclusion and exclusion criteria

Inclusion criteria:

1. Subjects: patients with schizophrenia
2. Interventions: Combination of TCM and Silibinin in the treatment of NAFLD
3. Control: Silibinin alone
4. Outcome indicators: Total effective rate, changes in the levels of triglycerides (TG), total cholesterol (TC), alanine aminotransferase (ALT), aspartate aminotransferase (AST), gamma glutamyl transpeptidase (GGT) and TCM syndrome score.
5. Study design: Randomized controlled trial (RCT)

Exclusion criteria:

Duplicate publications; studies for which full text was not available or for which data extraction was not possible; studies using animal studies; reviews and systematic reviews.

## 3. Search strategy

In this meta-analysis, we searched PubMed, Embase, Cochrane Library, CNKI, Wanfang, CQVIP and CBM databases from the establishment of the databases to Aug 2023. The mesh terms used were: “nonalcoholic Fatty Liver Disease” AND “Chinese medicine” “Chinese traditional medicine” “traditional Chinese medicine” “Integrated traditional Chinese and western medicine” “Combination of traditional Chinese and western medicine” AND “Silibinin” AND “Randomized Controlled Trial” “Randomized.” Specific search strategies in English are as follows: ((nonalcoholic Fatty Liver Disease[Title/Abstract]) AND (((((Chinese medicine[Title/Abstract]) OR (Chinese traditional medicine[Title/Abstract])) OR (traditional Chinese medicine[Title/Abstract])) OR (Integrated traditional Chinese[Title/Abstract] AND western medicine[Title/Abstract])) OR (Combination of traditional Chinese[Title/Abstract] AND western medicine[Title/Abstract]))) AND (Randomized[Title/Abstract])

## 4. Literature screening and data extraction

Two researchers conducted the search for information, the screening of information and the capture of information respectively. Any questions or disagreements were made after consultation with a third party. Information was collected by author, year, study design, number of cases and outcome indicators.

## 5. Literature quality assessment

Two researchers carried out separate independent evaluations of the quality of the literature, using the Review manager 5.3^[12]^ software risk assessment tool to evaluate the included literature according to random sequence generation, allocation concealment, blinding, whether research results were blinded to review, completeness of outcome data, using the Cochrane Risk Assessment Scale, and gender, selection of reported research outcomes, other biases, etc, and in cases of disagreement, through discussion or consultation with third parties. The Meta-analysis was performed in accordance with the relevant items in the Preferred Reporting Items for Systematic Reviews and Meta Analysis (PRISMA) statement.^[13]^

## 6. Data synthesis and statistical analysis

Data were analyzed using STATA 15.1.^[14]^ Weighted Mean differences (WMD) with 95%CI were used as continuous variables. I^2^ was used to evaluate cell heterogeneity. If the test for heterogeneity was *P* ≥ .1 and I^2^ ≤ 50%, homogeneity between studies was indicated and the studies were analyzed together using a fixed effects model; if *P* < .1 and I^2^ > 50%, significant heterogeneity between this group was indicated; if there was a difference, the source of the difference was identified using sensitivity analysis (We did a sensitivity analysis to exclude each of these trials one by one, and then did a combined analysis of the remaining trials). If the differences were still large, the random-effects model was used or the results of the combined study were discarded in favor of descriptive analysis. Publication bias was analyzed using funnel plots.

## 7. Results

### 7.1. Literature search results

**Figure 1.**
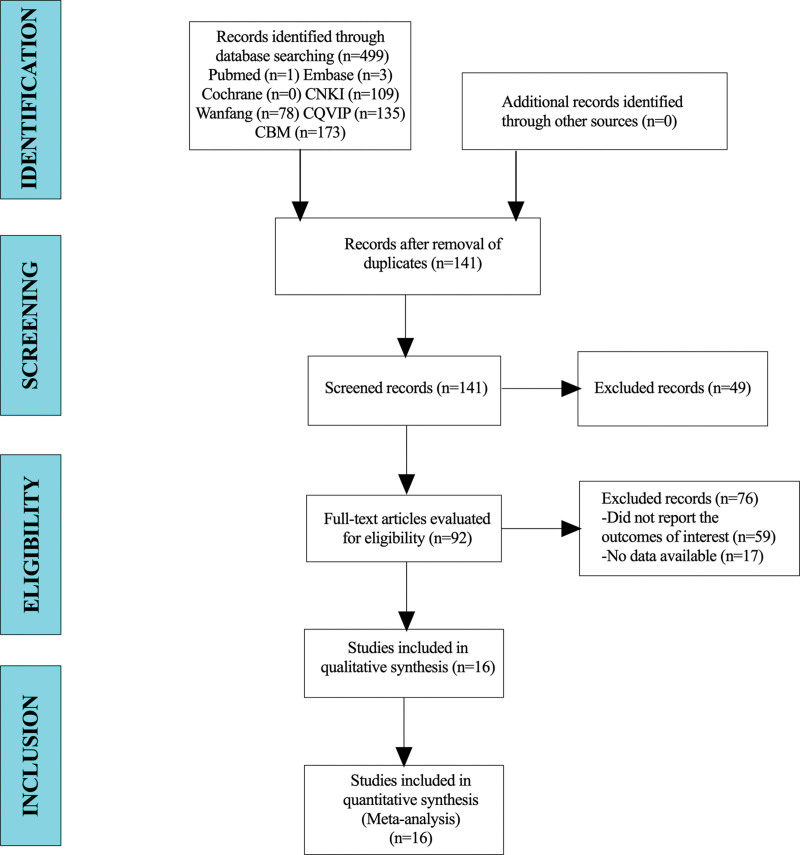
Flow diagram for selection of studies.

A total of 499 articles were collected for this study. After excluding duplicate trials, 141 patients were included in this study. A total of 92 articles were identified after reading their titles and abstracts. Finally, 16 studies were included in the meta-analysis (Fig. 1).

## 8. Baseline characteristics and quality assessment of the included studies

**Figure 2.**
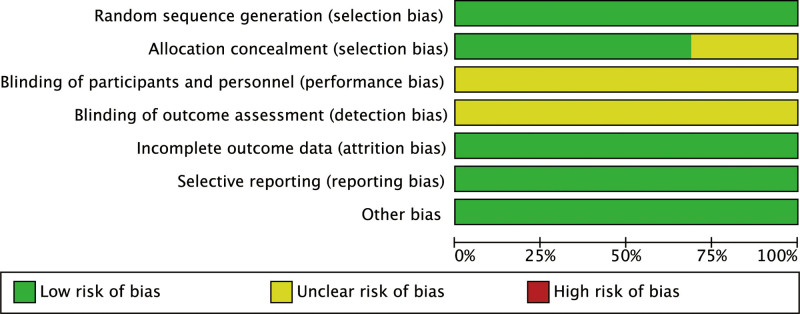
Risk of bias graph.

**Figure 3.**
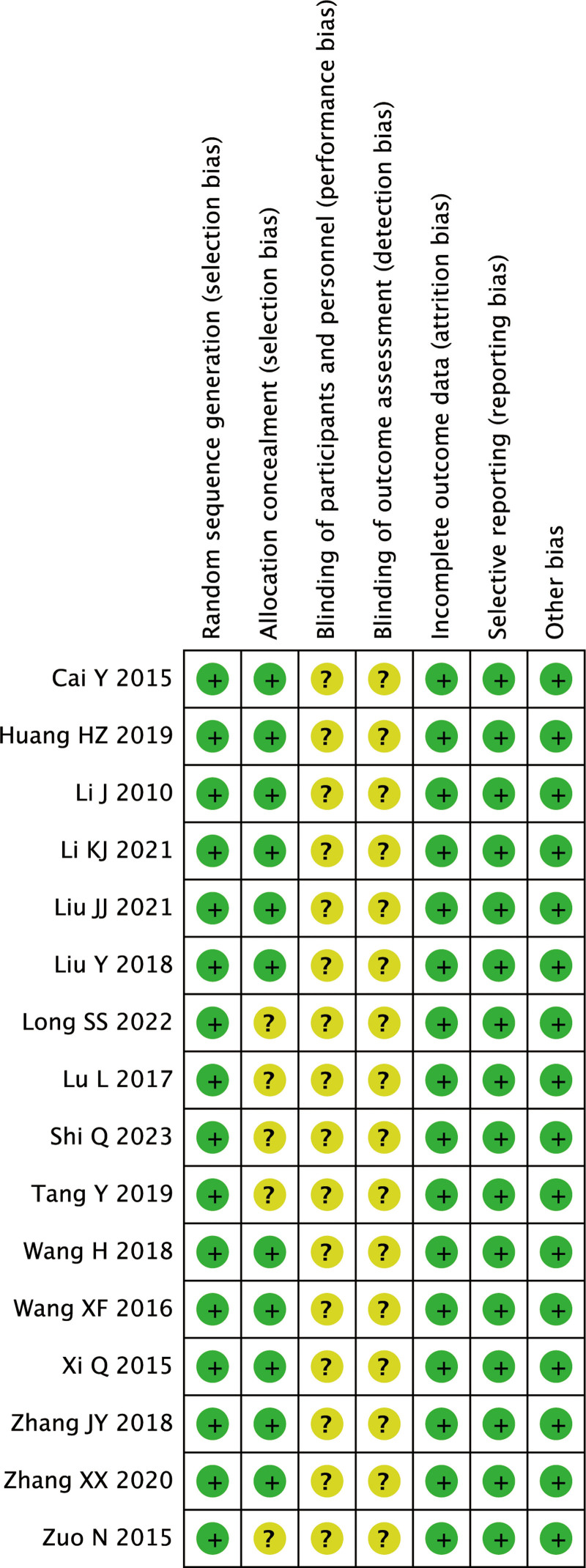
Risk of bias summary.

16 RCT studies were included in this meta-analysis. The sample size ranged from 48 to 120, with a total of 1335 patients, including 669 in the Combined treatment group and 384 in the Silibinin group. The age distribution of patients in the combined treatment group was 18 to 65, and the age distribution of patients in the control group was 20 to 66, suggesting that the ages were comparable (Table 1). The quality assessment results of the 16 RCTs are shown in Figures 2–3. The findings showed that all studies included in this review described the formation of random sequences. Eleven articles described “Allocation concealment,” but none described whether blinding was used.

**Table 1:** Baseline characteristics and quality assessment of the included studies.

Author	Yr	Study design	Sample size		Sex (male/female)		Age		Duration of disease (yr)		Measurements	
			Combined treatment group	Control group	Combined treatment group	Control group	Combined treatment group	Control group	Combined treatment group	Control group	Combined treatment group	Control group
Haizhen^[15]^	2019	RCT	54	54	30/24	28/26	41.4 ± 11.5	41.9 ± 11.3	2.3 ± 0.7	2.5 ± 0.7	Baogan Jiangzhi Decoction + Silibinin Capsules	Silibinin Capsules
Zhang et al^[16]^	2020	RCT	30	30	24/6	23/7	48.5 ± 9.4	50.2 ± 7.2	3.3 ± 1.2	3.1 ± 1.0	Baogan Jiedu Decoction + Silibinin Capsules	Silibinin Capsules
Hui and Wei^[17]^	2018	RCT	50	50	27/23	24/26	45.9 ± 12.0	44.6 ± 11.7	/	/	Danshao Shugan Granules + Silibinin Capsules	Silibinin Capsules
Xiaofeng et al^[18]^	2016	RCT	40	40	32/8	31/9	44.9	45.2	1	1	Gan Zhi Ping Granule + Silibinin Capsules	Silibinin Capsules
Ying et al^[19]^	2018	RCT	25	25	15/10	14/11	34.5 ± 6.6	33.8 ± 7.0	5.2 ± 1.6	5.3 ± 2.0	Huanglian Jiedu Decoction + Silibinin Capsules	Silibinin Capsules
Jie et al^[20]^	2010	RCT	33	32	20/13	18/14	36.8 ± 11.7	38.3 ± 12.8	/	/	Jianpi Shugan Granules + Silibinin Capsules	Silibinin Capsules
Nan et al^[21]^	2015	RCT	42	42	21/21	20/22	36.7 ± 8.6	37.3 ± 11.7	/	/	Jianpi Shugan Granules + Silibinin Capsules	Silibinin Capsules
Zhang et al^[22]^	2018	RCT	46	46	9/37	8/38	52.9 ± 9.4	53.0 ± 9.4	4.5 ± 1.9	4.4 ± 2.0	Jiangzhi Ligan Decoction + Silibinin Capsules	Silibinin Capsules
Shi^[23]^	2023	RCT	40	40	28/12	30/10	40.2 ± 3.1	41.3 ± 2.8	1.3 ± 0.2	1.4 ± 0.1	Qinggan Huashi Huoxue Decoction + Silibinin Capsules	Silibinin Capsules
Qi et al^[24]^	2015	RCT	45	45	30/15	31/14	47.8 ± 9.6	43.6 ± 12.0	1.4 ± 1.2	1.7 ± 1.3	Shugan Jianpi Decoction + Silibinin Capsules	Silibinin Capsules
Lin and Qing^[25]^	2017	RCT	24	24	12/12	12/12	45–65	44–66	/	/	Self-Proposed TCM Prescription + Silibinin Capsules	Silibinin Capsules
Panyu et al^[26]^	2021	RCT	54	54	25/29	26/28	53.2 ± 8.3	52.8 ± 8.7	1.5 ± 0.2	1.5 ± 0.2	Xiaozhi Hugan Decoction + Silibinin Capsules	Silibinin Capsules
Yue et al^[27]^	2015	RCT	35	35	28/7	30/5	18–56	20–54	/	/	Xingqi Huatan Method + Silibinin Capsules	Silibinin Capsules
Yan and Jie^[28]^	2019	RCT	56	54	45/11	40/14	25–65	20–66	/	/	Quzhi Prescription + Silibinin Capsules	Silibinin Capsules
Shuan-Shuang et al^[29]^	2022	RCT	60	60	28/32	36/24	47.4 ± 11.8	46.7 ± 12.2	2.0 ± 1.2	2.2 ± 1.2	Self-Proposed Xiaopi Huatan Granules + Silibinin Capsules	Silibinin Capsules
Kaijie et al^[30]^	2021	RCT	35	35	23/12	21/14	42.4 ± 4.6	43.2 ± 5.1	4.1 ± 1.7	4.2 ± 1.9	Self-Proposed TCM Prescription + Silibinin Capsules	Silibinin Capsules

RCT = randomized controlled trial, TCM = traditional Chinese medicine.

### 8.1. Results of meta-analysis

#### 8.1.1. Total effective rate.

**Figure 4.**
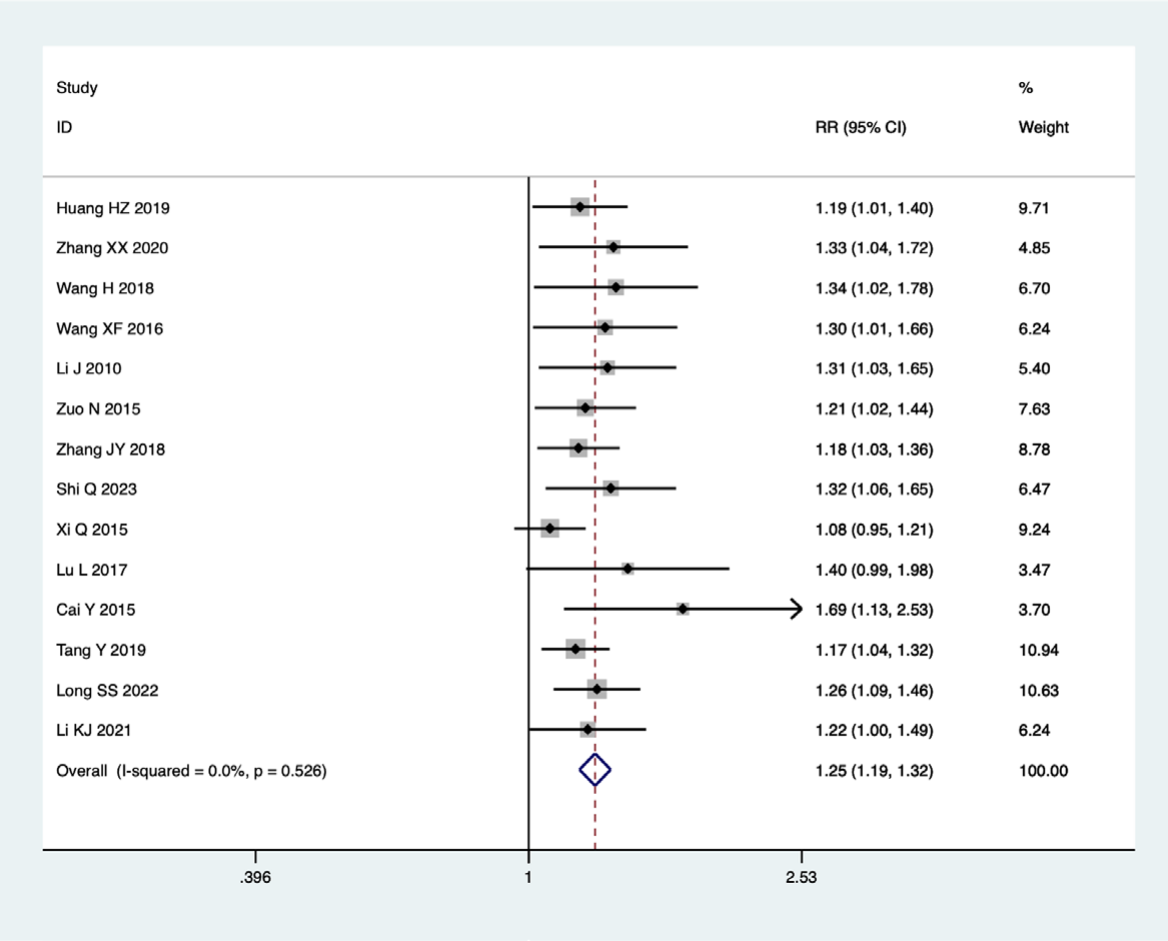
Comparing the differences in total effective rate between combined treatment group and Silibinin group in the treatment of NAFLD. NAFLD = non-alcoholic fatty liver disease.

Fourteen studies were conducted to compare the total effective rate of TCM combined with Silibinin and Silibinin alone in the treatment of NAFLD. As no significant heterogeneity was observed (I²=0.0%, *P* = .526), a fixed-effect model was employed for the meta-analysis. The findings indicated that the total effective rate of combined treatment group was significantly higher than that of Silibinin alone (RR = 1.25, 95% CI: 1.19 to 1.32, *P* = .000) (Fig. 4).

#### 8.1.2. TC levels.

**Figure 5.**
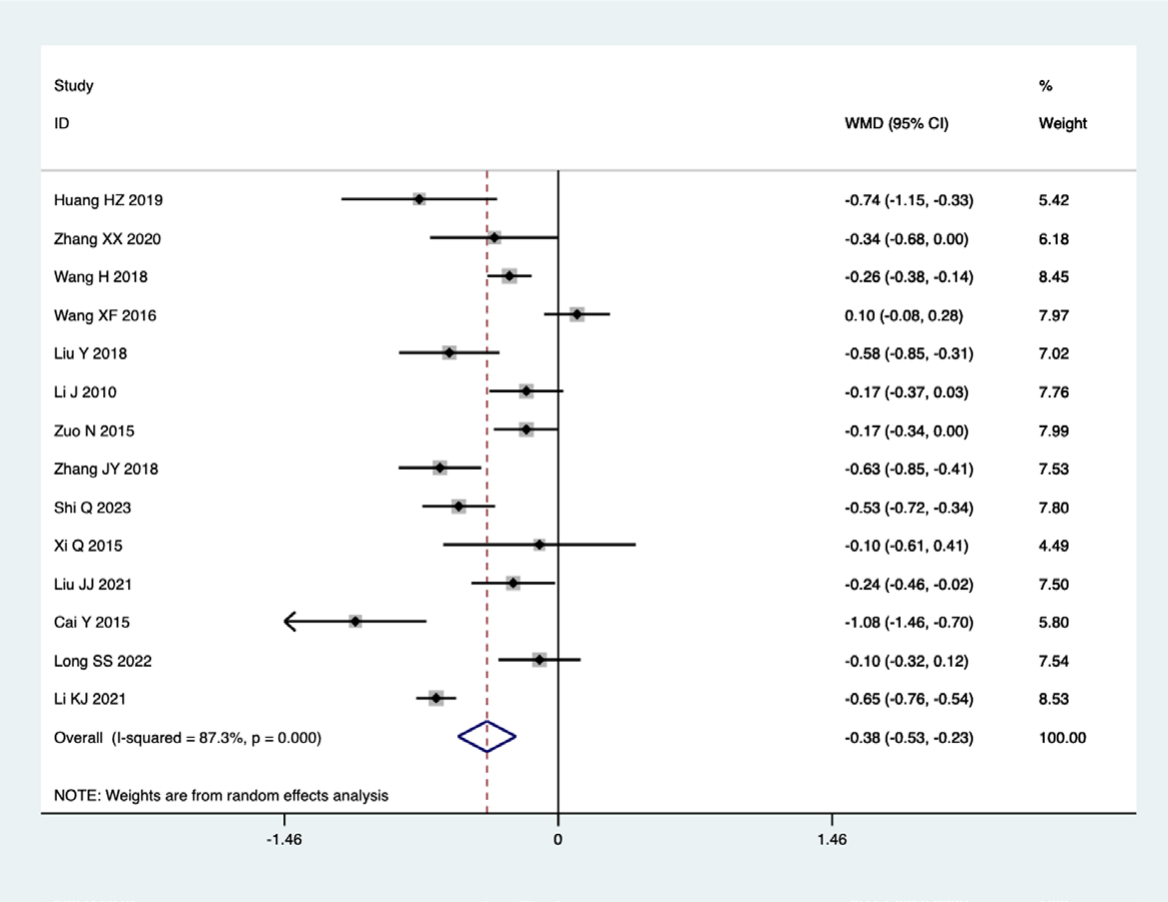
TC levels in the combined treatment group and the Silibinin group after treating NAFLD. NAFLD = non-alcoholic fatty liver disease.

Fourteen studies conducted comparisons of TC levels in the treatment of NAFLD with TCM in combination with Silibinin versus Silibinin alone. Since there was significant heterogeneity (I^2^ = 87.3%, *P* = .000), a random-effect model was used for the meta-analysis. The summarized results indicated that after treating NAFLD with a combination of TCM and Silibinin, the TC levels were significantly lower than those observed with Silibinin alone (WMD = −0.38, 95% CI: −0.53 to −0.23, *P* = .000) (Fig. 5).

#### 8.1.3. TG levels.

**Figure 6.**
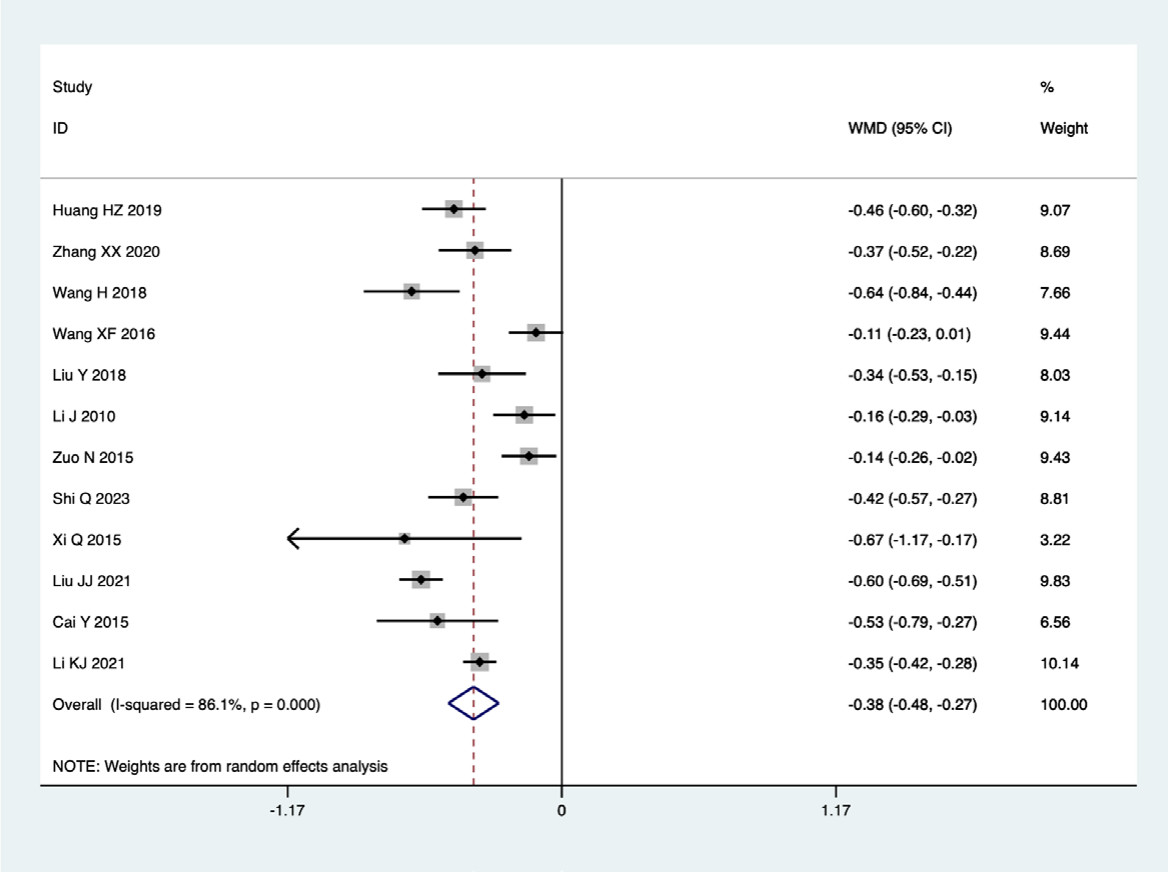
TG levels in the combined treatment group and the Silibinin group after treating NAFLD. NAFLD = non-alcoholic fatty liver disease.

Twelve studies conducted comparisons of TG levels in the treatment of NAFLD with TCM in combination with Silibinin versus Silibinin alone. Since there was significant heterogeneity (I^2^ = 86.1%, *P* = .000), a random-effect model was used for the meta-analysis. The summarized results indicated that after treating NAFLD with a combination of TCM and Silibinin, the TG levels were significantly lower than those observed with Silibinin alone (WMD = −0.38, 95% CI: −0.48 to −0.27, *P* = .000) (Fig. 6).

#### 8.1.4. ALT levels.

**Figure 7.**
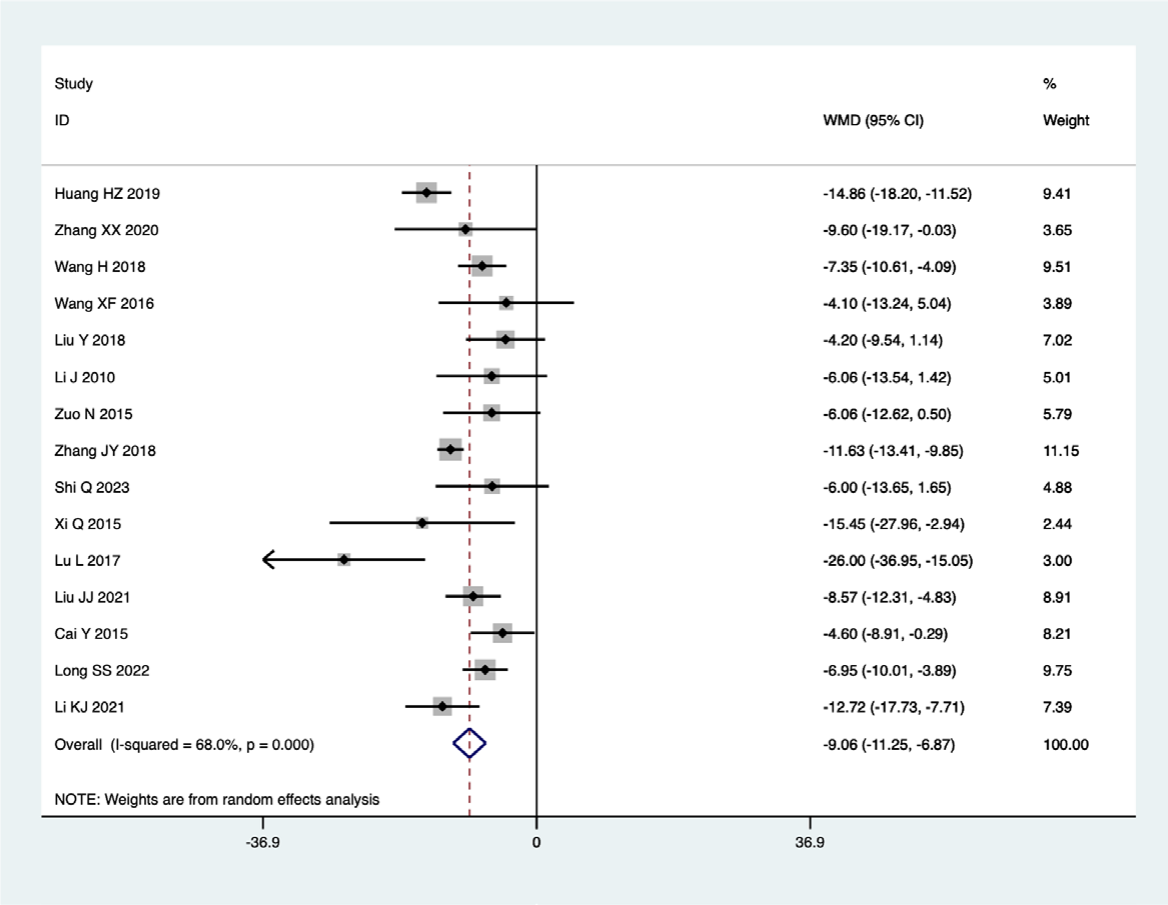
ALT levels in the combined treatment group and the Silibinin group after treating NAFLD. ALT = alanine aminotransferase, NAFLD = non-alcoholic fatty liver disease.

Fifteen studies conducted comparisons of ALT levels in the treatment of NAFLD with TCM in combination with Silibinin versus Silibinin alone. Since there was significant heterogeneity (I^2^ = 68.0%, *P* = .000), a random-effect model was used for the meta-analysis. The summarized results indicated that after treating NAFLD with a combination of TCM and Silibinin, the ALT levels were significantly lower than those observed with Silibinin alone (WMD = −9.06, 95% CI: −11.25 to −6.87, *P* = .000) (Fig. 7).

#### 8.1.5. AST levels.

**Figure 8.**
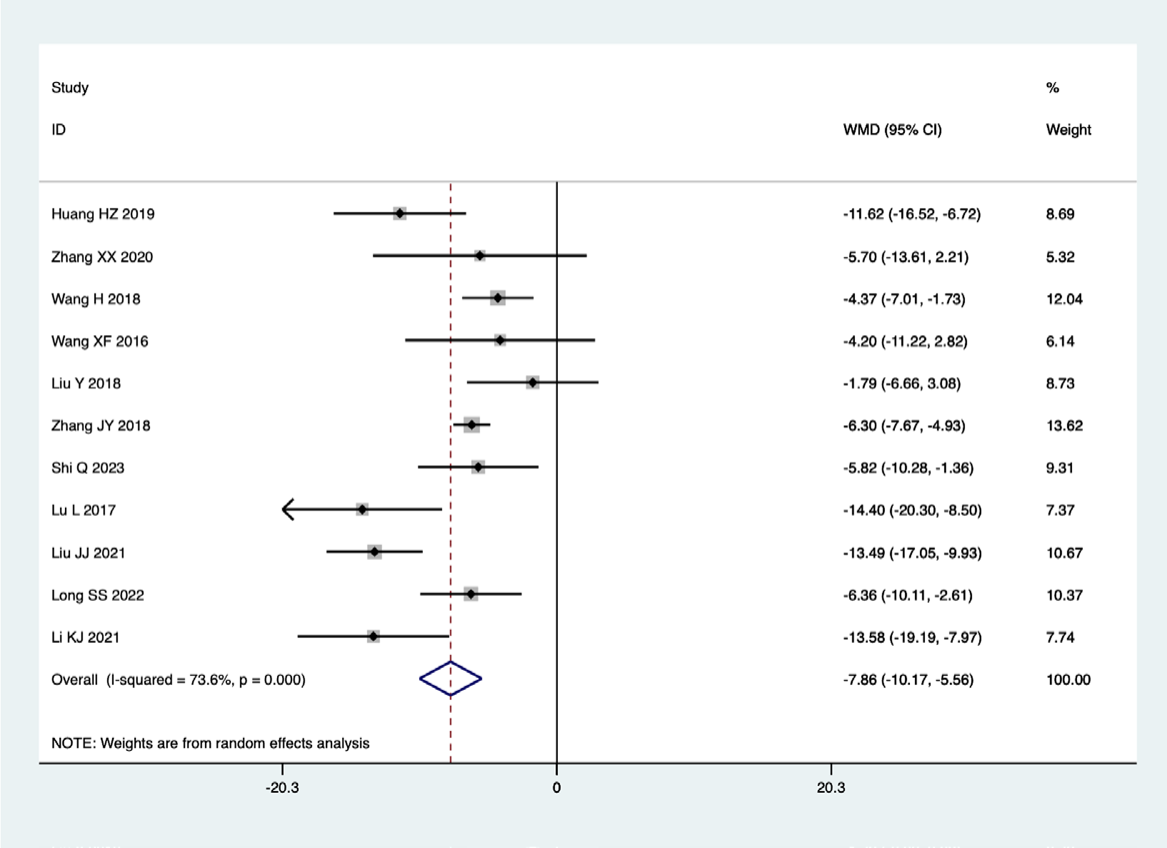
AST levels in the combined treatment group and the Silibinin group after treating NAFLD. AST = aspartate aminotransferase, NAFLD = non-alcoholic fatty liver disease.

Fifteen studies conducted comparisons of AST levels in the treatment of NAFLD with TCM in combination with Silibinin versus Silibinin alone. Since there was significant heterogeneity (I^2^ = 68.0%, *P* = .000), a random-effect model was used for the meta-analysis. The summarized results indicated that after treating NAFLD with a combination of TCM and Silibinin, the AST levels were significantly lower than those observed with Silibinin alone (WMD = −9.06, 95% CI: −11.25 to −6.87, *P* = .000) (Fig. 8).

#### 8.1.6. GGT levels.

**Figure 9.**
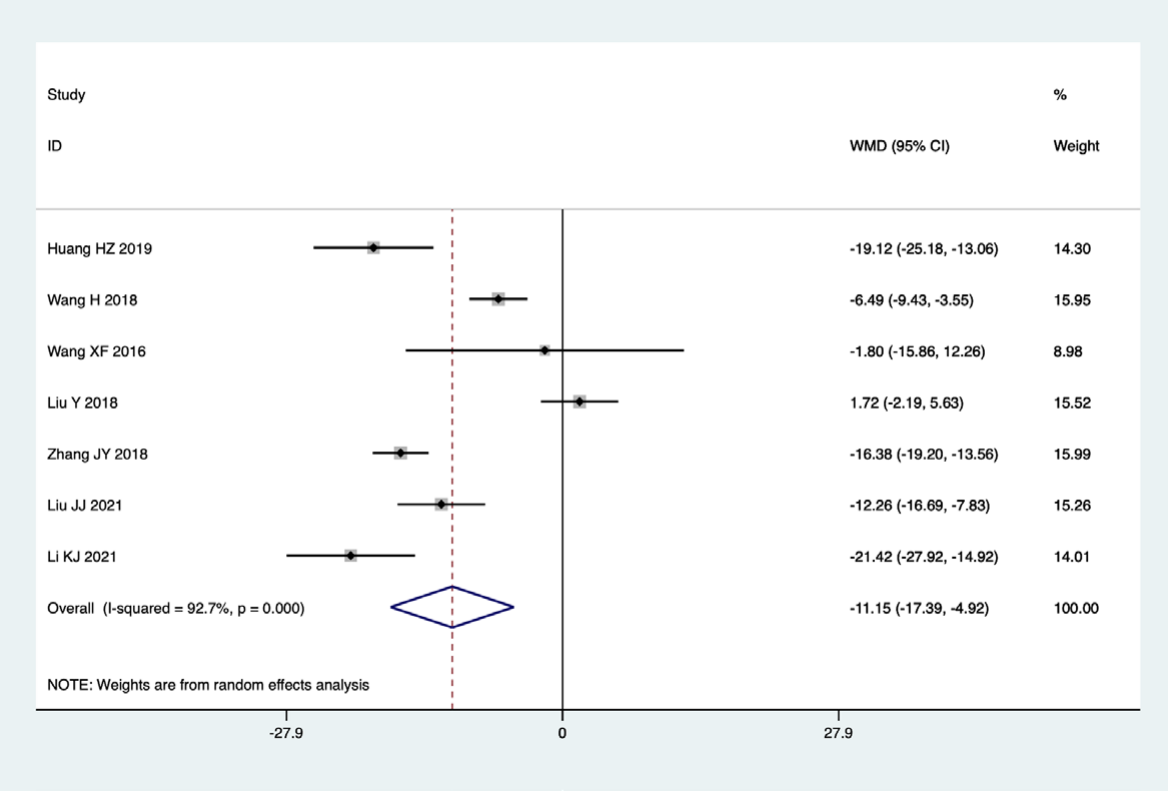
GGT levels in the combined treatment group and the Silibinin group after treating NAFLD. GGT = gamma glutamyl transpeptidase, NAFLD = non-alcoholic fatty liver disease.

Seven studies conducted comparisons of GGT levels in the treatment of NAFLD with TCM in combination with Silibinin versus Silibinin alone. Since there was significant heterogeneity (I^2^ = 92.7%, *P* = .000), a random-effect model was used for the meta-analysis. The summarized results indicated that after treating NAFLD with a combination of TCM and Silibinin, the GGT levels were significantly lower than those observed with Silibinin alone (WMD = −11.15, 95% CI: −17.39 to −4.92, *P* = .000) (Fig. 9).

#### 8.1.7. TCM syndrome score.

**Figure 10.**
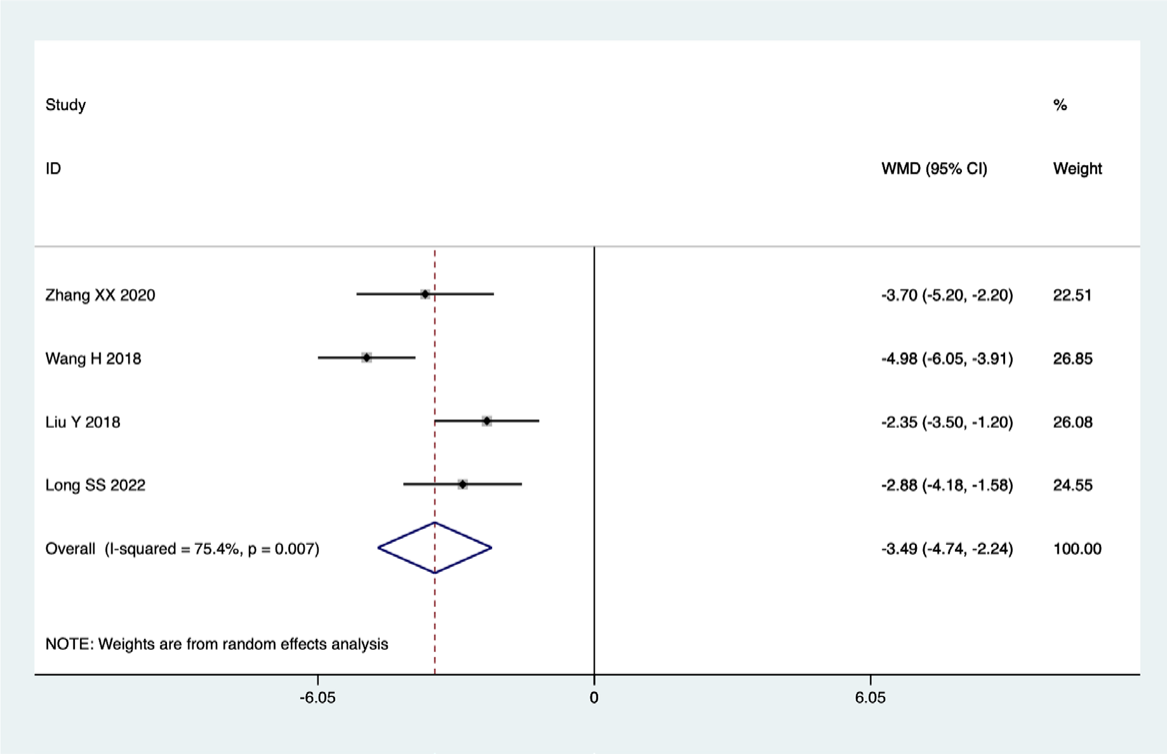
TCM syndrome score in the combined treatment group and the Silibinin group after treating NAFLD. NAFLD = non-alcoholic fatty liver disease, TCM = traditional Chinese medicine.

Four studies conducted comparisons of TCM syndrome score in the treatment of NAFLD with TCM in combination with Silibinin versus Silibinin alone. Since there was significant heterogeneity (I^2^ = 75.4%, *P* = .007), a random-effect model was used for the meta-analysis. The summarized results indicated that after treating NAFLD with a combination of TCM and Silibinin, the TCM syndrome score were significantly lower than those observed with Silibinin alone (WMD = −3.49, 95% CI: −4.74 to −2.24, *P* = .000) (Fig. 10).

### 8.2. Sensitivity analysis

We did a sensitivity analysis to exclude each of these trials one by one, and then did a combined analysis of the remaining trials. By doing a Meta-analysis, we found that all the Meta-analyses did not have much effect on the results of the Meta-analysis, indicating that the results of the Meta-analysis were stable and reliable.

### 8.3. Publication bias

**Figure 11.**
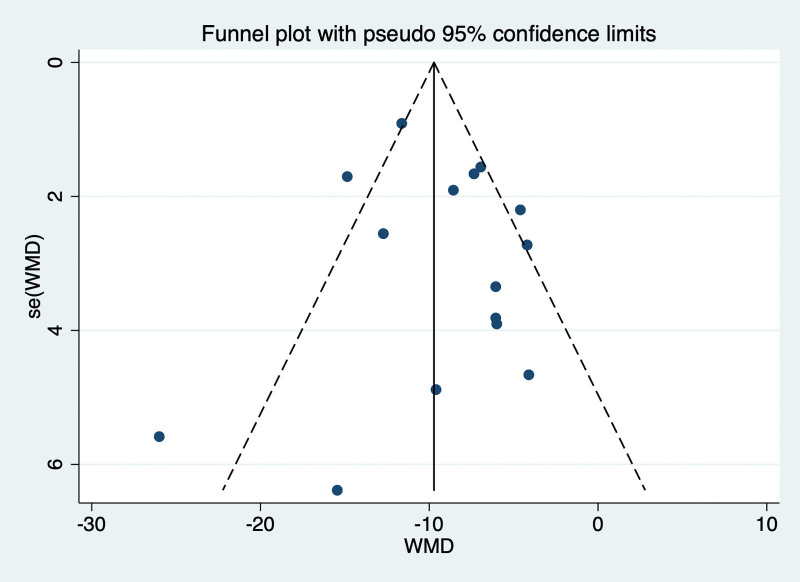
Funnel plot for evaluating the publication bias of this meta-analysis.

The funnel plot drawn in this study is as follows, and it can be seen that the funnel diagram is basically symmetrical. The *P* value obtained by Egger test was .524, indicating that no significant publication bias was found in this paper (Fig. 11).

## 9. Discussion

According to the clinical symptoms and characteristics of patients, NAFLD can be classified within the realm of TCM as conditions related to “fat-dampness,” “hypochondriac pain,” “accumulation,” and “liver stagnation.” Its pathological location is the liver, with pathological factors such as phlegm-dampness, damp-heat, qi stagnation, and blood stasis. These various pathological factors can intersect, leading to a complex presentation that may include both deficiency and excess conditions. It can be differentiated into pattern types such as liver depression with spleen deficiency, phlegm-dampness obstruction, damp-heat retention, or phlegm-blood stasis intermingling, among others. For clinical treatment of NAFLD with concurrent liver damage, the main therapeutic approach involves behavioral interventions, liver protection, and managing enzymes, while also focusing on blood sugar and lipid control.^[31]^ Silibinin capsules have antioxidative and anti-inflammatory properties. They work by inhibiting the generation of fatty acids, reducing the accumulation of lipids and peroxidation in the liver. Moreover, they improve mitochondrial function and enhance lipid exchange between the interior and exterior of cells, thereby alleviating cellular lipid stress. These actions have been shown to be clinically effective and safe.^[32]^

This study summarizes clinical randomized controlled trials of TCM in combination with Silibinin for the treatment of NAFLD. By employing various software for analysis, assessing bias risks, and evaluating evidence quality, it aims to provide evidence-based medicine data for the combined treatment of NAFLD using both traditional Chinese and Western medicine. The results indicate that, regardless of treatment duration, administration methods, medication forms, or geographical distribution, the total effective rate of TCM in combination with Silibinin is significantly superior to the control group, with statistically significant differences. This suggests a clinical advantage for TCM in conjunction with Silibinin. TCM offers the advantage of targeting multiple mechanisms, making it an important source for the development of new approaches and drugs for NAFLD. However, due to the lack of repetition in the TCM methods used in the included literature, it is unclear which specific TCM approach is superior.

In terms of observed indicators, it was found that liver function markers (ALT, AST, GGT), lipid metabolism markers (TG, TC), and TCM syndrome scores showed more significant improvements in the combined treatment of traditional Chinese and Western medicine. In fact, during the data collection process for this study, it was noted that some researchers used fasting insulin, insulin index, leptin, adiponectin, and other parameters as observed indicators. However, due to limited available data, these parameters were eventually excluded from this study. NAFLD is closely associated with metabolic syndrome, type 2 diabetes, cardiovascular diseases, and colorectal tumors. Therefore, we need more evidence to support the inclusion of additional observational indicators. Mass spectrometry-based multi-omics studies are gaining popularity, and it is promising to obtain biomarkers for NAFLD from genomics, transcriptomics, proteomics, and metabolomics. The “gut-liver axis” has been shown to be of great significance in NAFLD, and iron death, closely related to lipid peroxidation, has also been implicated in the pathophysiology of NAFLD.^[33]^ Therefore, utilizing multi-omics, iron death, or studying the gut microbiota associated with NAFLD as observational indicators can help elucidate the mechanisms of traditional Chinese medicine in the context of NAFLD.

This study has certain limitations: Traditional Chinese medicine prescription and medication lacked standardization. Included literature primarily used classic formulas, clinical experience, or research on commercially available patent medicines as the basis for interventions. The diversity in intervention measures, along with variations in herbal flavors and dosages in traditional Chinese medicine compound components, made it challenging to compare results and interpret them consistently, which reduced the comparability between trials. Lack of follow-up records. None of the 16 RCTs included in this study had any follow-up data, preventing a deeper understanding of the prognosis of NAFLD when treated with traditional Chinese medicine in conjunction with Silymarin capsules.

## 10. Conclusion

Traditional Chinese medicine in conjunction with Silibinin capsules has shown significant efficacy in the treatment of NAFLD, improving clinical symptoms, blood lipid levels, and liver function. Furthermore, it is essential to engage in multi-omics research, investigate iron death, and explore the gut microbiota as potential observational indicators for the diagnosis and inclusion criteria. Conducting more high-quality clinical experiments is necessary to further validate these findings.

## Author contributions

**Conceptualization:** Xiang Zhang, Qiujun Zhou.

**Data curation:** Xiaoliang Jin, Qiujun Zhou.

**Formal analysis:** Xiang Zhang, Qiujun Zhou.

**Funding acquisition:** Zhenghao Jiang.

**Methodology:** Xiaoliang Jin.

**Software:** Xiaoliang Jin.

**Writing – original draft:** Xiang Zhang, Xiaoliang Jin, Qiujun Zhou.

**Writing – review & editing:** Zhenghao Jiang, Xiang Zhang, Xiaoliang Jin, Qiujun Zhou.

## References

[1] van Dijk AM Schattenberg JM Holleboom AG. Referral care paths for non-alcoholic fatty liver disease-Gearing up for an ever more prevalent and severe liver disease. United European Gastroenterol J. 2021;9:903–9. 10.1002/ueg2.12150 PMC8498396 34609086

[2] de Groot J Santos S Geurtsen ML. Risk factors and cardio-metabolic outcomes associated with metabolic-associated fatty liver disease in childhood. EClinicalMedicine. 2023;65:102248. 37855025 10.1016/j.eclinm.2023.102248 PMC10579278

[3] Chacon C Arteaga I Martinez-Escude A. Clinical epidemiology of non-alcoholic fatty liver disease in children and adolescents The LiverKids: study protocol. PLoS One. 2023;18:e0286586. 37831682 10.1371/journal.pone.0286586 PMC10575486

[4] Zhu S Wu Z Wang W. A revisit of drugs and potential therapeutic targets against non-alcoholic fatty liver disease: learning from clinical trials. J Endocrinol Invest. 2023. doi:10.1007/s40618-023-02216-y. 10.1007/s40618-023-02216-y 37839037

[5] Neuschwander-Tetri BA. Non-alcoholic fatty liver disease. BMC Med. 2017;15:45. 28241825 10.1186/s12916-017-0806-8 PMC5330146

[6] Cobbina E Akhlaghi F. Non-alcoholic fatty liver disease (NAFLD) - pathogenesis, classification, and effect on drug metabolizing enzymes and transporters. Drug Metab Rev. 2017;49:197–211. 28303724 10.1080/03602532.2017.1293683 PMC5576152

[7] Younossi Z Tacke F Arrese M. Global perspectives on nonalcoholic fatty liver disease and nonalcoholic steatohepatitis. Hepatology. 2019;69:2672–82. 30179269 10.1002/hep.30251

[8] Buzzetti E Pinzani M Tsochatzis EA. The multiple-hit pathogenesis of non-alcoholic fatty liver disease (NAFLD). Metabolism. 2016;65:1038–48. 26823198 10.1016/j.metabol.2015.12.012

[9] Bagherniya M Nobili V Blesso CN. Medicinal plants and bioactive natural compounds in the treatment of non-alcoholic fatty liver disease: a clinical review. Pharmacol Res. 2018;130:213–40. 29287685 10.1016/j.phrs.2017.12.020

[10] Gong P Long H Guo Y. Chinese herbal medicines: the modulator of nonalcoholic fatty liver disease targeting oxidative stress. J Ethnopharmacol. 1169;318:27. 10.1016/j.jep.2023.116927 37532073

[11] Cao Y Fang X Sun M. Preventive and therapeutic effects of natural products and herbal extracts on nonalcoholic fatty liver disease/nonalcoholic steatohepatitis. Phytother Res. 2023;37:3867–97. 37449926 10.1002/ptr.7932

[12] Yan C Xiong Y Chen L. A comparative study of the efficacy of ultrasonics and extracorporeal shock wave in the treatment of tennis elbow: a meta-analysis of randomized controlled trials. J Orthop Surg Res. 2019;14:248. 31387611 10.1186/s13018-019-1290-y PMC6683364

[13] Sarkis-Onofre R Catala-Lopez F Aromataris E. How to properly use the PRISMA Statement. Syst Rev. 2021;10:117. 33875004 10.1186/s13643-021-01671-z PMC8056687

[14] Zhu ZG Wang JR Pan XY. Efficacy of scraping therapy on blood pressure and sleep quality in stage I and II essential hypertension: a systematic review and meta-analysis. J Integr Med. 2023;S2095-4964:00097–3. 10.1016/j.joim.2023.11.006 38104001

[15] Haizhen H. Clinical study on baogan jiangzhi tang combined with silibinin for nonalcoholic steatohepatitis. New Chin Med. 2019;51:158–61.

[16] Zhang XX Yandong L Chuan Y. Decoction retention enema combined with silybin in the treatment of non-alcoholic steatohepatitis. Acta Chin Med Pharmacol. 2020;48:47–51.

[17] Hui W Wei Z. Clinical observation of 100 cases on “Danshao Shugan Granules”combined with “Silibinin capsules”in the treatment of humid heat and spleen deficiency type of nonalcoholic steatohepatitis. J Integrative Chin Western Hepatol. 2018;28:14–6.

[18] Xiaofeng W Wenjun M Xiaodan F. Ganzhiping granules for the treatment of non-alcoholic fatty liver disease. Jilin Tradit Chin Med. 2016;36:454–6.

[19] Ying L Xianzhong H Meiling X. Observation of the therapeutic effect of huanglian jiedu decoction combined with silibin capsule on non-alcoholic steatohepatitis with dampness-heat accumulation. Chin J Basic Med Tradit Chin Med. 2018;24:1258–61.

[20] Jie L Xinxing W Shugan J. Pill combined with Silybin Capsule in the treatment of non-alcoholic steatohepatitis: Clinical Observation. Chin J Tradit Chin Med Inform. 2010;17:68–9.

[21] Nan Z Huabin T Shugan J. Pill combined with silybin capsule for the treatment of non-alcoholic steatohepatitis. Northern Pharm. 2015;12:44–5.

[22] Zhang J Zhang Y Liu QQ. Effects of jiangligan decoction combined with silybin on liver function and blood lipid level in patients with non-alcoholic fatty liver disease. Modern Distance Educ Chin Med. 2023;21:107–10.

[23] Shi Q. Clinical observation of qinggan huoxue decoction and silybin capsule in the treatment of non-alcoholic fatty liver. Modern Distance Educ Chin Tradit Med. 2023;21:80–3.

[24] Qi X Yazhu L Chunrong S. Shugan Jianpi huoxue fang combined with Silibin capsule for Steatosis. Shaanxi J Tradit Chin Med. 2015;36:520–2.

[25] Lin L Qing W. Observation of the curative effect of silibinin combined with chinese medicine on 24 cases of non-alcoholic fatty liver disease. Inner Mongolia Tradit Chin Med. 2017;36:27.

[26] Panyu L Yuqing Y Miaoqing Y. Effects of Xiaozhihugan Decoction combined with silybin on glucose and lipid metabolism and liver function in patients with nonalcoholic fatty liver disease. World J Integrated Chin Western Med. 2021;16:1177–80.

[27] Yue C Chz Z Xueshu W. Clinical observation of treating non-alcoholic fatty liver with qi-eliminating phlegm. Chin Med Rev. 2015;21:83–5.

[28] Yan T Jie L. Observation on the curative effect of integrated Chinese and western medicine in the treatment of non-alcoholic steatohepatitis. J Practical Chin Med. 2019;35:454–5.

[29] Shuan-Shuang L Wei J Yan-nan D. Self-prepared xiaoxihuatan granule combined with silybin capsule in the treatment of non-alcoholic steatohepatitis (phlegm-dampness internal obstruction syndrome). Chinese Medicine Review 2022;28:66–70.

[30] Kaijie L Shujing Z Jian H. Efficacy of self-formulated Chinese medicine combined with silybin in the treatment of non-alcoholic fatty liver disease. J Hebei North Univ (Natural Science Edition). 2018;34:35–7.

[31] Junxiang L Jing C Yunliang W. Consensus opinion on the diagnosis and treatment of integrative chinese and western medicine in non-alcoholic fatty liver disease (2017). Chin J Integrative Chin Western Med Digest. 2017;25:805–11.

[32] Zhi-Peng Z Yan-Qiu M Zhi W. Research Progress on biological activity and mechanism of silybin and its derivatives. Chin Herbal Med. 2021;52:3717–24.

[33] Jayachandran M Qu S. Non-alcoholic fatty liver disease and gut microbial dysbiosis- underlying mechanisms and gut microbiota mediated treatment strategies. Rev Endocr Metab Disord. 2023;24:1189–204. 37840104 10.1007/s11154-023-09843-z

